# Effect of disturbance stimulation using a split-belt treadmill on a patient with cerebellar ataxia: a case report

**DOI:** 10.1186/s13256-023-03777-5

**Published:** 2023-02-19

**Authors:** Saho Myojin, Hiroyuki Yasumura, Jun Takashiba, Shu Morioka

**Affiliations:** 1 Chikamori Rehabilitation Hospital, 2-1 Nijyudaicho, Kochi, 780-0843 Japan; 2 Neurorehabilitation Research Center, Kio University, 4-2-2 Umaminaka, Koryo, Kitakatsuragi, Nara 635-0832 Japan

**Keywords:** Cerebellum, Ataxia, Split-treadmill, Gait

## Abstract

**Purpose:**

We present the case of a patient with cerebellar ataxia who was treated with walking practice using a split-belt treadmill with disturbance stimulation. The treatment effects were evaluated for improvements in standing postural balance and walking ability.

**Case presentation:**

The patient was a 60-year-old Japanese male who developed ataxia after cerebellar hemorrhage. Assessment was performed using the Scale for the Assessment and Rating of Ataxia, Berg Balance Scale, and Timed Up-and-Go tests. A 10 m walking speed and walking rate were also assessed longitudinally. The obtained values were fit into a linear equation (*y* = *ax* + *b*), and the slope was calculated. This slope was then used as the predicted value for each period relative to the pre-intervention value. After removing the trend of the value for each period relative to the pre-intervention value, the amount of pre- to post-intervention change for each period was calculated to verify the intervention effect. Furthermore, to verify the changes in gait over time, a three-dimensional motion analyzer was used to analyze the pre- and post-intervention gait five times, and the results were kinematically compared.

**Results:**

No significant pre- to post-intervention changes were observed in the Scale for the Assessment and Rating of Ataxia scores. Conversely, the Berg Balance Scale score, walking rate, and 10 m walking speed increased, and the Timed Up-and-Go score decreased in the B1 period, indicating a marked improvement from the predicted results based on the linear equation. For changes in gait determined using three-dimensional motion analysis, an increase in stride length was observed in each period.

**Conclusion:**

The present case findings suggest that walking practice with disturbance stimulation using a split-belt treadmill does not improve inter-limb coordination, but contributes to improving standing posture balance, 10 m walking speed, and walking rate.

## Background

Smooth and stable walking is achieved by coordinated control of multiple limbs and numerous muscle activities. The coordinated movements of both lower limbs that enable walking are output via the central pattern generator (CPG), a neural circuit that generates the gait rhythm in the spinal cord. However, controlling only the CPG when the external environment changes, such as uneven terrain or slopes, is difficult. The cerebellum plays a role in regulating locomotion while adapting to the environment, thus enabling humans to adapt to a variable environment and continue walking. This adaptation requires immediate postural responses to changes in the external environment, internal somatosensory perceptions, and anticipatory postural adjustment (APA) functions that precede the target movement.

The cerebellum plays an important role in postural control and motor coordination in response to error signals based on changes in the external environment. When the cerebellum is damaged, it is difficult to respond to disturbances based on environmental variables [1], thereby impairing predictive postural control [2], resulting in decreased standing postural balance and difficulty in stable walking [3].

The tied-belt treadmill intervention, with equal left and right belt speeds, has been developed to improve gait disturbance in patients with cerebellar injury [4, 5]. In particular, interventions such as stepping targets for visual obstacle avoidance, and projecting obstacles on a treadmill belt, have shown improvements in obstacle avoidance ability, and dynamic stability during obstacle avoidance. However, the effect of these interventions on improvements in standing postural balance and basic walking ability remain unclear.

Recently, a split-belt treadmill has been developed, which can change the environment and is expected to artificially create walking adaptation in humans. The split-belt treadmill, which allows independent modulation of the left and right belt velocities, is used clinically as a means of improving gait in stroke patients. In addition, it has shown to improve gait asymmetry in patients with hemiplegic stroke [6]. Asymmetrical movements of the left and right limbs become apparent in many patients with hemiplegic stroke. In particular, temporal asymmetry has been found to be strongly related to walking speed, affecting walking propulsion and walking efficiency [6–10].

Alternately, the split-belt treadmill can generate an error (perturbation) by changing the speed of the left and right belts. To adapt to the error, two processes are required: reactive adjustment, which is an immediate response, and predictive adjustment, which predicts and adapts to the subsequent error. It has been confirmed that the split-belt treadmill increases error signals, and increases the frequency of excitation of cerebellar Purkinje cells, while walking in an artificially altered environment [11]. In addition, a study using a split-belt treadmill in patients with cerebellar injury showed that reactive adjustment was possible [12–14]. Therefore, we hypothesized that walking practice using a split-belt treadmill, which can generate errors using disturbance stimuli, would contribute to the improvement of standing posture balance and walking ability. Thus, in this case study, a patient with ataxia after cerebellar injury underwent gait training using a split-belt treadmill, and the effectiveness of the training was examined using the ABAB design method. The novelty of this case study is the longitudinal evaluation of the effects of walking exercise using split treadmill training on a patient with ataxia.

## Case presentation

### Case

A 65-year-old Japanese man was receiving rehabilitation medicine such as physiotherapy for ataxia of the right upper and lower limbs and trunk due to cerebellar hemorrhage in the right hemisphere. Written informed consent was obtained from the patient for publication of this case report and any accompanying images. A copy of the written consent is available for review by the Editor-in-Chief of this journal.

### Course

**Fig. 1:**
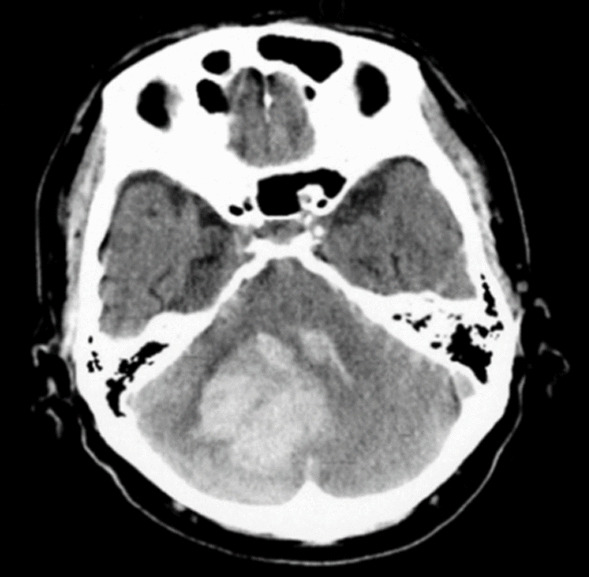
CT image of the head at onset. Hemorrhage in the right cerebellar peduncle and hemisphere (hemorrhage volume 37 mL). *CT* computed tomography

The patient was diagnosed with hypertensive cerebellar hemorrhage (Fig. 1) by a medical doctor in May 2019 using computerized tomography (CT) imaging, and underwent endoscopic hematoma removal on the day of onset. Diagnostic considerations were reviewed and agreed upon with the patient and his family before deciding whether or not to proceed with the examination. The main symptom was ataxia of the right upper and lower limbs and trunk. Acute physical therapy was started the next day, the patient was transferred to the recovery ward on the 34th day after surgery, and intervention with a split-belt treadmill was started on the 103rd day.

Prior to the intervention, objective assessment revealed that the Ueda 12-step hemiplegia function test was V-3 for the right lower extremity, the Scale for the Assessment and Rating of Ataxia (SARA) total score was 21.5, Berg Balance Scale (BBS) total score was 25 points, Timed Up-and-Go test (TUG) was 25 seconds, 10 m comfortable walking speed was 1.1 km per hour, and walking rate (steps per minute) was 106. For walking ability, walking with a walker required light to moderate assistance, while walking alone required constant trunk support and heavy assistance from a physical therapist. In addition, we examined eye movements using the International Cooperative Ataxia Rating Scale (ICARS) sub-item “evaluation of abnormal eye movements” [15]. The results showed that gaze-induced nystagmus was scored at 2 points; abnormal gaze movement, 1 point; and measurement disorder in eye movement, 2 points, indicating clear eye movement disorder. The Mini-Mental State Examination score (MMSE) was 20 points on admission and 28 points on discharge. There was no medical history and no falls during hospitalization.

## Intervention

**Fig. 2:**
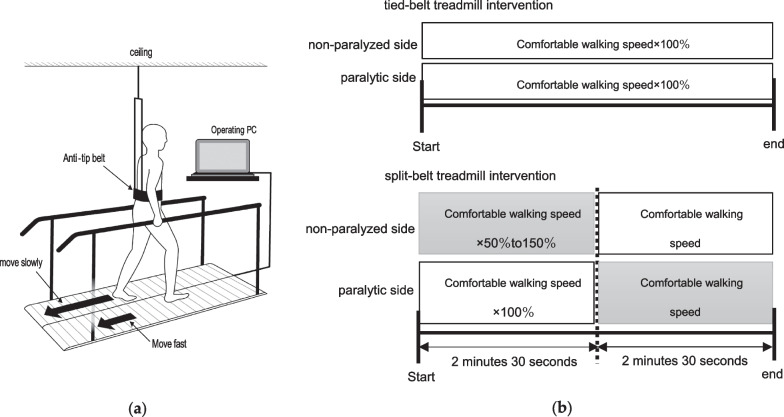
Intervention environment and methods. **a **Synchronous and asynchronous low-floor dual treadmill. The height and width of the handrails can be adjusted to appropriate positions, and the speed can be adjusted from 0.1 km at low-speed drive using an operating PC. The disturbance stimulus of the split-belt treadmill intervention modulates the belt speed on one side during walking and provides an unexpectedly disturbing stimulus to the posture. **b** Practice settings for tied-belt treadmill intervention and split-belt treadmill intervention. No disturbance stimuli were provided in the tied-belt treadmill intervention. In the split-belt treadmill intervention, the disturbance stimulus was given to one side at a speed within the range of 50–150% of the flatland walking speed. Upper extremity function training involved task-oriented exercises that combined joint movements such as shoulder flexion, elbow extension, and forearm rotation

Split-belt treadmill gait practice was conducted using a synchronous/asynchronous low-floor dual treadmill DLF-55 (Otake Root Factory, Morioka, Japan) (Fig. 2A). The belt speed in each period was the average of two measurements of the 10 m comfortable walking speed on level ground during each intervention period. The tied-belt treadmill intervention (A1 and A2) was conducted at 100% of the comfortable walking speed on level ground, and the split-belt treadmill intervention (B1 and B2) was conducted by increasing or decreasing the belt speed, within the speed range of 50–150% of the comfortable walking speed on level ground, and by providing disturbance stimuli that unexpectedly disturbed the posture. Exercises were conducted for a total of 30 minutes, with three sets of 5 minutes each, and a rest period between each set to recover from subjective fatigue. The disturbance stimulus of the split-belt treadmill was provided within a set speed range using an operating personal computer. Intervention involved randomly modulating belt velocity on the non-paralyzed side from the start until 2 minutes and 30 seconds, and then the belt velocity on the paralyzed side was randomly modulated for a subsequent 2 minutes and 30 seconds. Simultaneously, the opposite side without disturbance stimulus was set at 100% of the comfortable walking speed on level ground (constant speed). Duration of the disturbance stimulus was set to between 2 and 10 seconds, and this duration varied with each new stimulus (Fig. 2B). Intervention period for the tide-belt treadmill intervention and the split-belt treadmill intervention was 2 weeks each, and the interventions were conducted twice alternately for a total of 8 weeks. The amount of assistance was maintained at a minimum to prevent falling. In addition, muscle strengthening exercises for the trunk and lower limbs were performed as exercise therapy for 50 minutes a day, with appropriate breaks, as well as assisted walking exercises on level ground wearing a fall prevention belt in a room with a road surface.

## Measurements

The following items were measured a total of five times: once at the start of the intervention and four times at the end of each 2 week period during the 8 week study duration. SARA was used to assess ataxia. BBS, TUG, walking rate, and 10 m comfortable walking speed were used to assess standing postural balance and walking ability. For TUG, a 40-cm-high chair was used and the starting posture was sitting in the chair, leaning lightly on the backrest, with hands on the thighs. The patient was asked to rise from the chair at a signal, walk around a target 3 m away at the maximum walking speed, and sit on the chair again. The time required to walk around the target was measured.

In the 10 m walking test, the time required to walk at a comfortable walking speed along a 10 m walking path was measured. Measurements were recorded twice, and the average value was calculated as the walking speed. The values of BBS, TUG, walking rate, and 10 m comfortable walking speed, which were calculated before the intervention and at the end of each period, were fit to a linear function equation of *y* = *ax* + *b*, and the slope was calculated. This slope is shown as the predicted value for each measurement item; from this predicted value, we first removed the trend of each period’s value relative to the pre-intervention value. From the trend-eliminated values, the amount of change before and after the intervention for each period was further calculated, and the value was shown as the effect of the intervention for each period.

For the three-dimensional motion measurement, reflective markers were attached to the patient’s body at a total of 12 locations: the center of the acromion on both sides, the iliac crest, the one-third position from the greater trochanter on the straight line connecting the superior anterior iliac spine and the greater trochanter, the midpoint of the anterior–posterior diameter of the lateral femoral epicondyle, lateral malleolus, and the fifth metatarsal head. A three-dimensional motion analysis system, Kinema Tracer (Kissei Comtec Co., Ltd.), and four small Charge Coupled Device cameras were synchronized to record treadmill walking, with one handrail grasped, for 20 seconds at a sampling frequency of 60 Hz. The speed of the treadmill walking during the measurement was set by measuring the 10 m comfortable walking speed twice on level ground, and the average of the two speeds was calculated as the comfortable walking speed on level ground. These movement measurements were performed a total of five times: once at the start of the intervention, and four times at the end of each 2-week period of the intervention. The walking rate was calculated from the number of steps taken during 20 seconds of measurement.

A fall prevention belt was worn during the measurement, and the amount of assistance was minimized to the extent that the patient did not fall. The walking rate and time factors were calculated as the percentage of time spent in the stance phase, swing phase, and bipedal support phase, and the distance factors were calculated as stride length, step length, and stride distance.

## Results

SARA and BBS scores are presented in Table 1. In the subordinate items of SARA, the standing position improved from 5 points before the intervention, to 4 points in the A1 period, and 3 points in the B1 period. The sitting position improved from 2 points before the intervention to 1 point in the A1 period. No other changes were observed in gait, speech disturbance, or limb ataxia. In the B1 period, the BBS showed improvement in “transfer, closed-leg stance, forward reach of upper limbs, picking up objects from the floor, turning back over the right and left shoulders, 360° rotation, standing in the joint position, and one-legged stance.”

**Table 1:** Changes in SARA and BBS sub-items over time

	Pre-intervention	A1	B1	A2	B2
SARA					
Gait	6	6	6	6	6
Standing	5	4	3	3	3
Sitting position	2	1	1	1	1
Speech disorder	3	3	3	3	3
Finger chase	1	1	1	1	1
Nose–finger test	1.5	1.5	1.5	1.5	1.5
Fast alternative hand movement	1.5	1.5	1.5	1.5	1.5
Heel–shin slide	1.5	1.5	1.5	1.5	1.5
Overall score	21.5	19.5	18.5	18.5	18.5
BBS					
Sitting to standing	3	3	3	4	4
Standing unsupported	3	4	4	4	4
Sitting unsupported	4	4	4	4	4
Standing to sitting	2	3	3	3	3
Transfers	2	2	3	3	3
Standing with eyes closed	3	3	3	4	4
Standing with feet together	2	2	3	3	3
Reaching forward with outstretched arm	3	3	4	4	4
Retrieving objects from floor	2	2	3	3	3
Turning to look behind	1	2	3	3	4
Turning 360°	0	1	3	3	3
Placing alternate foot on stool	0	0	0	0	0
Tandem standing	0	1	2	2	2
Standing on one leg	0	0	2	1	2
overall score	25	30	40	41	43

*SARA* Scale for the Assessment and Rating, *BBS* Berg Balance Scale

**Fig. 3:**
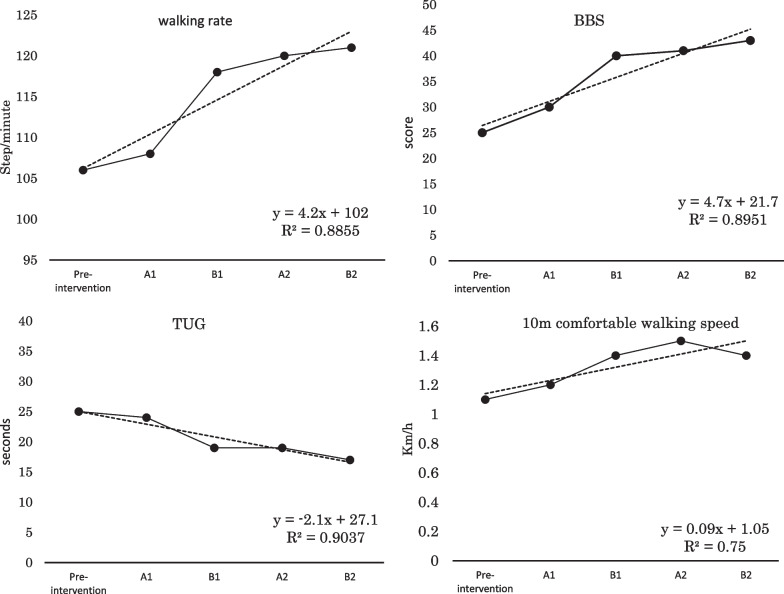
Measured values and prediction equations for walking rate, BBS, TUG, and 10 m comfortable walking speed. The dotted line graph shows the predicted values for each intervention period obtained by fitting the actual values for each intervention period into a linear function equation, and the line graph shows the actual values. Walking rate, BBS, and 10 m comfortable walking speed increased significantly from A1 to the end of B1 in each measurement item, and decreased in TUG. In addition, pre-intervention to the end of A1, B2 to the end of A2, and A2 to the end of B2 periods showed only a slight increase or decrease and no characteristic changes. *BBS* Berg Balance Scale, *TUG* Timed Get-Up and Go

Measured values and predicted equations for the BBS, TUG, walking rate, and 10 m comfortable walking speed are shown in Fig. 3. For the amount of change before and after each intervention period, the following items showed significant changes after trend removal: BBS increased to 5.3 from A1 to the end of B1, TUG decreased to −2.9 from A1 to the end of B1, walking rate increased to 5.8 from A1 to the end of B1, and 10 m comfortable walking speed increased to 0.11 km/hour from A1 to the end of B1. Alternately, no characteristic changes were found from pre-intervention to the end of A1, B2 to the end of A2, and A2 to end of B2.

**Fig. 4:**
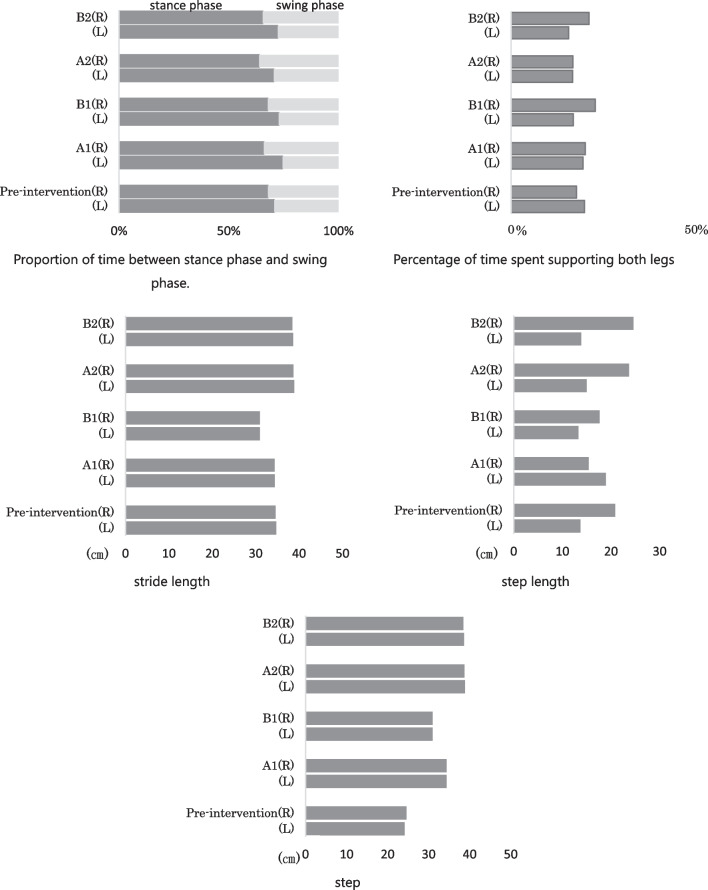
Results of changes in time and time factor proportions. In terms of the time factor of the gait cycle, the difference between the right and left sides of the B1 and B2 phases of the gait cycle was larger than the difference between that in the A1 and A2 phases of the bilateral leg support phase. In the distance factor, stride length was shortened only in the B1 period and extended in the A2 period and later. The step length of the right lower extremity increased over time only after the A1 period, and the stride distance increased in the B1 and A2 periods

Results of the three-dimensional motion analysis are presented in Fig. 4. In terms of the time factor of the gait cycle, the difference between the right and left sides of the two legs was larger in the B1 and B2 phases than in the A1 and A2 phases. For the distance factor, the stride length was shortened only in the B1 period and extended after A2 period. In terms of step length, only the right lower extremity showed extension over time from stage A1 onward, and the stride distance increased in stages B1 and A2.

Regarding oculomotor disturbance, gaze-induced nystagmus on the ICARS showed improvement from 2 points to 1 point; however, the scores of abnormalities in pursuit eye movements, and measurement disturbance in eye movements, did not change; the oculomotor disturbance persisted.

In terms of walking ability, walking with a walker improved to light assistance, and walking alone improved to light or moderate assistance, where the physical therapist needed to support the trunk to prevent a fall. The patient was discharged on postoperative day 165. Indoor mobility was independent in a wheelchair, and walking with a walker required monitoring and light assistance. This section may be divided by subheadings. It should provide a concise and precise description of the experimental results, their interpretation, as well as the experimental conclusions that can be drawn.

## Discussion and conclusions

A patient with cerebellar ataxia was treated with treadmill walking with disturbance stimulation using a split-belt treadmill. The results based on the single-case design method showed improvements in the BBS, TUG, walking rate, and walking speed.

Based on the overall SARA score, the patient had moderate to mild ataxia. Previous studies have shown that moderate to mild cases can adapt to disturbances and that people with cerebellar injury have residual reactive coordination to disturbances [12–14, 16]. Therefore, it is considered that patients with moderate to mild ataxia can respond to disturbances; thus, the treatment was sufficiently adaptable in this case. In addition, the present results suggest that the split-belt treadmill intervention may improve reactive postural coordination and contribute to the improvement of postural balance and walking ability, even in the presence of residual impairments in anticipatory postural control and ataxia.

Another study confirms that walking with an artificially altered environment using a split-belt treadmill increased the error signal and frequency of excitation of cerebellar Purkinje cells [11]. Postural responses are necessary to maintain body stability when disturbances are applied during exercise [17]. While walking on a split-belt treadmill, reactive postural adjustment is required because of the addition of disturbances based on velocity changes. Therefore, we believe that the BBS scores increased, which contributed to the improvement of postural balance ability. Given the improvement in walking speed, we speculate that the improvement of postural balance using a split-belt treadmill combined with high-sped repetitions contributed to the increase in walking rate. In addition, TUG has been shown to correlate with walking speed, walking rate, and BBS [18, 19]; thus, we infer that the improvements in postural balance and walking ability were also related in our results.

The functional recovery curve of motor function in hemiplegic stroke patients generally shows a significant improvement within 3 months after the onset of stroke [20], and is similar for patients with cerebellar hemorrhage or infarction [21]. In this case, the intervention was performed more than 3 months after the onset of the disease, and significant improvements in balance and walking ability were observed in the B2 phase. Therefore, the confounding effect of spontaneous recovery was minimized.

Given the amount of change shown by the linear function equation and the results over time, the effects of the split-belt treadmill on postural balance and walking ability were not significant after the A2 period. Gait speed is strongly related to temporal asymmetry [8]. In this case, the temporal asymmetry did not improve according to the time and distance factors by the three-dimensional motion analysis, and the inter-limb coordination disorder persisted as evaluated using the SARA. In other words, the degree of improvement in walking speed may have been affected by the left–right asymmetry in the gait cycle and residual inter-limb coordination disorder due to ataxia. Moreover, previous studies have shown that the walking rate of men aged 65–69 years is 112.8 steps per minute [22]. In the present case, the walking speed was higher than the average walking speed of men of the same age at the end of the B1 period, so it is possible that the ceiling effect occurred at the end of the B1 period in this case only, and that the effect of the disturbance stimulus did not appear after that. In contrast, TUG includes motor elements of standing up from, or sitting down in, a chair and walking in a straight line towards a target object, and changing direction. Previous studies have shown that vision plays a role in gait, perceiving distant information, and fine-tuning gait in a predictive manner [23]. Therefore, we infer that this case did not show any effect after the A2 period due to diplopia.

The lack of significant improvements in the BBS and SARA sub-items, such as climbing stairs, standing in the joint position, standing on one leg, and walking on one leg, can be attributed to the fact that this study focused on improving postural balance and walking ability only during walking. Thus, there may have been a discrepancy between the expected effects of the split-belt treadmill intervention and the present study, and it remains unclear whether split-belt treadmill gait is transferable to other sub-items of the BBS and SARA—there is scope for future studies to explore this further.

In terms of stride length, it was shortened in stage B1, and extended in stage A2 and later. This may be due to the control of increasing the number of rotations (pitch) of the lower limbs in the B1 period, and the change to greater mechanical control using the lower limbs after the A2 period. However, in terms of step length, only the right lower limb was prolonged after the A2 stage. The prolonged stance phase time on the left side may be due to the compensatory reliance on the left lower limb, which was non-ataxic, and to obtain forward propulsion, resulting in greater left–right asymmetry. As the patient also had diplopia and limb ataxia, we hypothesized that the enlargement of the gait interval was compensatory in order to ensure stability of the standing posture balance during walking.

This case study suggests that the clinical intervention of split-treadmill may contribute to the improvement of standing postural balance disorder in cerebellar disease. However, we were not able to examine predictive postural control (anticipatory postural adjustments, etc.) in gait, and we could not determine whether the split-belt treadmill could contribute more to improving either reactive or predictive adjustment, due to the occurrence of errors (perturbations). Furthermore, the effect of the split-belt treadmill may depend on the ability to perceive changes in the speed of the belt [24, 25]. Therefore, the effects may differ depending on the degree of sensory disturbance. The above is a limitation of this case report, and it needs to be examined with a larger population.

A patient with moderate to mild ataxia due to cerebellar injury was provided gait training using a split-belt treadmill, and its effects were evaluated. Post-intervention, standing postural balance and walking ability improved; however, there was no clear improvement in the inter-limb coordination disorder. These results suggest that gait training with a split-belt treadmill may contribute to the improvement of walking ability by improving postural balance in patients with residual ataxia.

## Data Availability

All data used were obtained from the patient’s medical records in our hospital archives.
